# The Role of Multi-Sensor Measurement in the Assessment of Movement Quality: A Systematic Review

**DOI:** 10.1007/s40279-023-01905-1

**Published:** 2023-09-12

**Authors:** T. Alexander Swain, Melitta A. McNarry, Adam W. H. Runacres, Kelly A. Mackintosh

**Affiliations:** 1 Applied Sports, Technology, Exercise and Medicine Research Centre (A-STEM), Swansea University, Swansea, UK; 2 Institute of Sport, Manchester Metropolitan University, Manchester, UK

## Abstract

**Background:**

Movement quality is typically assessed by drawing comparisons against predetermined movement standards. Movements are often discretely scored or labelled against pre-set criteria, though movement quality can also be evaluated using motion-related measurements (e.g., spatio-temporal parameters and kinematic variables). Wearable technology has the potential to measure and assess movement quality and offer valuable, practical feedback.

**Objectives:**

A systematic approach was taken to examine the benefits associated with multi-sensor and multiple wearable-device usage, compared with unimodal applications, when assessing movement quality. Consequently, this review considers the additional variables and features that could be obtained through multi-sensor devices for use in movement analyses. Processing methods and applications of the various configurations were also explored.

**Methods:**

Articles were included within this review if they were written in English, specifically studied the use of wearable sensors to assess movement quality, and were published between January 2010 and December 2022. Of the 62,635 articles initially identified, 27 papers were included in this review. The quality of included studies was determined using a modified Downs and Black checklist, with 24/27 high quality.

**Results:**

Fifteen of the 27 included studies used a classification approach, 11 used a measurement approach, and one used both methods. Accelerometers featured in all 27 studies, in isolation (*n* = 5), with a gyroscope (*n* = 9), or with both a gyroscope and a magnetometer (*n* = 13). Sampling frequencies across all studies ranged from 50 to 200 Hz. The most common classification methods were traditional feature-based classifiers (*n* = 5) and support vector machines (SVM; *n* = 5). Sensor fusion featured in six of the 16 classification studies and nine of the 12 measurement studies, with the Madgwick algorithm most prevalent (*n* = 7).

**Conclusions:**

This systematic review highlights the differences between the applications and processing methods associated with the use of unimodal and multi-sensor wearable devices when assessing movement quality. Further, the use of multiple devices appears to increase the feasibility of effectively assessing holistic movements, while multi-sensor devices offer the ability to obtain more output metrics.

**Supplementary Information:**

The online version contains supplementary material available at 10.1007/s40279-023-01905-1.

## Key Points

**Table:** 

Wearable technology has been widely used in research to measure and classify matters relating to movement quality using an array of machine-learning and sensor-fusion methods.
Multiple multimodal sensor devices appear to be more effective than single multimodal sensor devices when assessing holistic movements.
Devices containing multiple sensors offer the ability to obtain more output metrics than those featuring just one sensor.

## Introduction

Movement quality is a historically overlooked component of physical activity and exercise monitoring outside of elite sports [1]. Whilst wearable technology has become an integral part of modern lifestyles, wearable movement tracker outputs are almost exclusively focused on movement quantity [2]. However, wearable technology also has the potential to measure and assess movement quality by facilitating specific and contextualised feedback [3]. Indeed, this is advantageous in the reduction of injury risk, given that poor quality movement is widely recognised as a contributor to injury [4–9]. Additionally, better movement quality is recognised to enhance life-expectancy by increasing motivation and confidence to engage in physical activity [10], and skill proficiency is fundamental for optimal athletic performance [11–13]. Furthermore, the development of motor competence across a wide array of motor skills in young people is an essential foundation for overall health throughout the lifecourse [10, 14]. Nonetheless, there remains an unmet demand surrounding the assessment of, and feedback regarding, movement quality, which could benefit greatly from the capabilities of wearable devices [14].

While the definition of movement quality is open to interpretation and likely context-specific, Venek et al. [15] offer a perspicuous, generalised definition of movement quality as “the degree to which replications of the original movements can be performed in comparison to either an expert or professional, or to a defined performance of an exercise” [15]. Further expanding on this definition, in clinical settings, movement quality may be assessed by comparing pathologically influenced movements against healthy controls, or normative data [16, 17]. Consequently, assessing movement quality necessitates a predetermined standard against which comparisons can be made, with discrete scoring or labelling systems often employed to distinguish between good and bad movements, different skill levels, or to highlight specific movement discrepancies [15]. Moreover, movement quality may also be evaluated using motion-based measurements, for example, spatio-temporal parameters and kinematic variables [1], if baseline information exists to draw comparisons against.

Bardid et al. [14] provide an overview of the wide range of options currently available in the context of assessing motor competence in children and adolescents, identifying a spectrum from which appropriate assessment tools can be selected depending on the application and criteria. However, it is postulated that there could be a large degree of applicability of this spectrum for assessing movement quality, beyond the confines of motor development in children and adolescents. Traditionally, movement-quality assessments have frequently been subjectively conducted by experts, such as physiotherapists, teachers, and/or trained assessors [15, 18–20]. However, modern technologies make it feasible to conduct objective assessments [1], and support individuals in the absence of a movement expert [21]. Camera-based technologies such as optical motion capture [22, 23] and depth cameras [22, 24, 25] are commonly used in the assessment of movement quality due to an array of advantages [22, 24, 25], though such methods are also associated with significant limitations [20, 22, 24, 25]. Wearable technology, however, may provide an affordable, practical, and efficient alternative for assessing movement quality [14, 20, 21, 26, 27], with the potential for automated feedback using numerical data or visualisations [28]. Currently, there are many commercially available wearable devices used to evaluate movement, including unimodal sensors, such as accelerometers, gyroscopes, and magnetometers [29–31], or multimodal sensor devices, for example, an inertial measurement unit (IMU) [32–35]. In addition, multiple multimodal sensors can be utilised, where they are positioned at different anatomical locations to provide additional and integrated outputs [27, 36]. Sensor combinations may enable a broader picture of movements, which would be valuable when assessing movement quality [1, 32].

Congruent with many subjective assessments [37–39], data obtained by technology may be utilised to categorise movement characteristics, using either binary or multi-class classification methods [20]. A binary classification approach is typically utilised to distinguish between a competent or non-competent movement; that is, whether or not an individual has demonstrated movement proficiency based on the criteria of a predetermined standard. A multi-class classification approach, however, adds a degree of specificity by highlighting specific characteristics [20]. The existing literature suggests that the performance of classification algorithms relative to others depends on many factors, such as the movement performed, sensor positioning, and the parameters considered [40–42]. Indeed, the accuracy of any output from a wearable device can be influenced by the hardware utilised, with sampling frequency being a key factor that needs to be optimised to achieve maximum performance [40, 43, 44]. Nevertheless, it is recognised that the use of additional sensors and wearable devices would enable the capture of a broader dataset with more measurable outputs, which would theoretically enable a more in-depth, and possibly accurate, classification by having a greater range of classifier inputs to select from [45]. Sensors are also commonly applied to directly measure specific motion characteristics, including kinematics such as acceleration, velocity, and displacement [46]. However, it is also possible to capitalise on concepts such as sensor fusion to estimate orientations and angles when multiple types of sensors are available within a device [45, 47, 48].

Previous reviews have provided insights into the potential use of wearable technology to widely assess movement quality [15, 20, 21, 49]. Most recently, a scoping review was conducted that provided a timely update on the array of technology-based measurement methods available for assessing movement quality, though the review only sought to provide a broad overview of technology usage in sport over a 5-year period [15]. Moreover, there is a lack of consideration in abovementioned reviews of the potential benefits of concurrent sensor usage, either within the same multimodal sensor unit, or using multiple devices, when compared to unimodal alternatives [15, 20, 21, 49]. The current systematic review, therefore, sought to highlight any additional benefits that could be deduced when concurrently applying multi-sensor devices, and indeed multiple wearable devices, for the assessment of movement quality, contextualised around sporting and clinical applications. Consequently, this review also investigated the additional variables, and indeed features, that could be obtained through multi-sensor devices compared to unimodal sensors for use in movement analyses. Finally, this systematic review aimed to distinguish between the processing methods and applications of multi-sensor wearable devices in comparison to unimodal sensors when assessing movement quality.

## Methods

The protocol was developed to implement a systematic approach in accordance with guidelines provided by the Preferred Reporting Items for Systematic Reviews and Meta-Analyses (PRISMA) [50, 51]. A Population Intervention Comparison Outcome (PICO) approach to the development of the systematic review framework was also employed. Details of the protocol for this systematic review were registered on PROSPERO (ID: CRD42020222587).

### Search Strategy

An initial electronic literature search was completed between November and December 2020, with additional searches conducted to incorporate any additional relevant publications up to December 2022. The searches sought to identify published materials indexed in the following five databases: MEDLINE, ACM Digital Library, IEEE Xplore, SPORTDiscus, and Scopus. Initial keywords ‘movement’, ‘quality’, ‘wearable’ and ‘human’ were identified as suitable group headings for an expanded search of other related search terms and synonyms, with a Boolean search strategy implemented thereafter (Table 1; Online Supplemental Resource (OSR) 1). The search strategy utilised keywords in lieu of subject headings to broaden the search. Search terms were sought from within the title, abstract and listed keywords for each publication.

**Table 1:** Boolean search strategy

General	Specific search terms
Movement	(movement* OR motion OR "human mechanics" OR kinematics OR biomechanics OR locomot* OR "motor skill*" OR gait OR ambulat*)
	AND
Quality	(quality OR proficien* OR competen* OR performance OR abilit*)
	AND
Wearable	(wearable* OR sensor* OR multi-sensor* OR multisensor* OR "inertial measurement unit*" OR IMU$ OR acceleromet* OR gyroscop* OR magnetomet* OR detector*)
	AND
Human	human* OR men OR women OR child* OR people OR adolescent$ OR teenagers OR athlete*

An asterisk (*) expanded the search to include all terms beginning with the letters preceding the asterisk. A dollar sign ($) expanded the search to include a single additional letter in the position of the dollar sign. The dollar sign was substituted with a question mark (?) when searching ACM Digital Library due to the criteria for this database

### Literature Screening and Selection

Articles retrieved from the electronic databases were initially stored using Mendeley referencing software (Mendeley Desktop, Version 1.19.8) before uploading to Rayyan [52] for screening. The PRISMA flowchart [50] was utilised to record the process of screening and selection. Duplicates arising from the use of multiple databases were identified within Rayyan and removed. Initially, titles and abstracts were screened for full-text review according to the inclusion and exclusion criteria (Table 2). The screening process was conducted by two authors (TAS and AWHR) and was blinded [52]; that is, each author screened the articles independently, with discrepancies revealed on conclusion of the screening phase. Inter-rater agreement was assessed at each stage of the screening process using kappa scores [53]. All articles were screened by the first author (TAS), while another researcher (AWHR) screened 10% [54, 55]. It is reasonable when undertaking large reviews for two reviewers to initially screen a small percentage of studies and discuss variation in the interpretation of the inclusion criteria [55]. In doing so, this aids consistency when the remaining studies are screened by a single reviewer for inclusion within the full-text screening phase [55]. A kappa score of 0.955, an almost perfect agreement [53], was observed during the title and abstract screening, after which the discrepancies among the dual-screened studies were resolved through discussion between the two reviewers. All full texts of the remaining articles were then screened against the inclusion and exclusion criteria by the same two reviewers, again independently under blind conditions. A weak agreement was initially observed, generating a kappa score of 0.491. However, following discussions to clarify the inclusion criteria, any conflicts were discussed until a consensus was reached. Where the two reviewers could not agree (*n* = 2), the discrepancies were discussed with a third independent reviewer (KAM) until a consensus was reached, subsequently resulting in a kappa score of 1.

**Table 2:** Study inclusion and exclusion criteria

*Inclusion criteria*
Wearable sensors were used for the purpose of assessing movement quality in medical and sporting applications
The wearable sensors were used to assess fine or gross motor skills
Human participants irrespective of age
The wearable devices were attached to individuals directly, or to manually operated equipment (i.e. sports equipment)
Articles were published between January 2010 and December 2022
Peer-reviewed articles published in the English language
Experimental and analytical observational studies
*Exclusion criteria*
Descriptive and qualitative studies
Systematic reviews and literature reviews
Non-peer-reviewed literature
Other unsuitable resources such as conference presentations, expert opinion, and grey literature
Studies that feature particularly varied or irregular movements, or sequential combination movement patterns (i.e., multiple discrete movement patterns performed transitionally in series)
Studies where wearable outputs were not assessed

### Data Extraction

Following screening, data were extracted from the included full texts by the lead author (TAS) and tabulated within a customised data extraction form. The data extraction form was subsequently reviewed by another author (MAM); where discrepancies were identified, the data extraction form was revised until both authors reached a consensus.

### Quality Assessment

Two authors (TAS and AWHR) evaluated the quality of the included studies to determine the risk of bias. The primary author (TAS) assessed the quality of all studies, while the other author (AWHR) assessed the quality of 14 of the 27 included studies, approximately 50%, in accord with Pai and McGrady [56, 57]. A modified version of the Downs and Black [58] checklist was selected as it can be used to assess the methodological quality of both randomised controlled trials and non-randomised studies. Specifically, the modifications made to the Downs and Black checklist [58], which were based on other systematic reviews synthesising the use of wearable technology [21, 49], ensured specificity and, therefore, relevance to the included studies. The criteria were accompanied by a rating system developed to categorise study quality as low (≤ 33.3%), moderate (33.4–66.7%), and high (≥ 66.8%; OSR 2). The limits of each scoring category were adopted from other systematic reviews focused on the use of wearable technology to measure and assess movement [21, 49]. Eighteen items were included in the checklist, with each item rated between zero and two (0 = not present, 1 = limited detail and 2 = good detail). Inter-rater reliability was calculated using kappa scores, with 0.8 identified as the minimum acceptable inter-rater agreement [53]. Initially, the kappa score for risk of bias was 0.273, indicative of fair agreement [53]. Discrepancies centred around study design, eligibility criteria, and the reliability of equipment, primarily due to each author’s interpretation of the checklist questions. The two authors discussed each point until a consensus was reached, resulting in a kappa score of 1. Subsequently, the remaining studies were reassessed based on the agreed interpretation of each quality assessment checklist item.

Given the broad inclusion criteria and subsequent methodological differences in the included studies, a narrative synthesis was conducted. The methodological approach is covered, with particular focus on the identification of sensor features, the application of sensors and the obtained data, and the techniques by which movement quality was assessed. Data processing and analysis methods, including sensor-fusion algorithms, machine-learning techniques and biological modelling, were of particular interest. The findings of the included studies were assessed, specifically focusing on the comparison between unimodal and independent uses of sensors and devices, and more systemic approaches, where the data obtained from multiple sensors and devices were concurrently utilised and integrated.

## Results

**Fig. 1:**
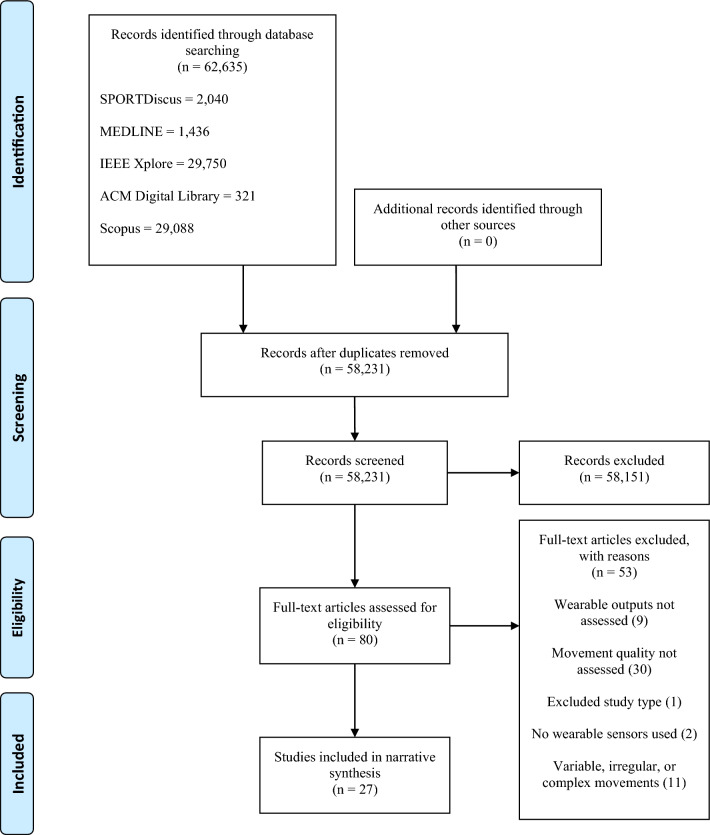
PRISMA flowchart [50]

A total of 62,635 titles were obtained across the five databases, with 58,231 titles remaining after the removal of duplicates. Following the screening of 80 full-texts, 27 articles were included in the final review (Fig. 1). For the quality assessment, 24 of the included studies were deemed to be of high quality (score > 66.8%), with three identified as moderate quality (score 33.4–66.7%; Table 3).

**Table 3:** Quality assessment for included studies

Study	1	2	3	4	5	6	7	8	9	10	11	12	13	14	15	16	17	18	Total score (out of 36)	Percentage score (%)	Quality category
Ahmadi et al. (2015) [59]	2	2	1	1	1	0	2	1	2	2	2	2	2	1	1	2	2	0	26	72.2	High
Beange et al. (2019) [60]	2	2	2	1	0	0	2	2	2	2	1	2	2	2	2	2	2	2	30	83.3	High
Bisi et al. (2017) [61]	2	2	2	1	1	0	2	2	2	2	2	2	2	2	1	2	2	2	31	86.1	High
Caporaso and Grazioso (2020) [62]	2	2	2	2	1	0	2	2	2	2	0	2	2	2	2	2	2	0	29	80.6	High
Cortesi et al. (2017) [63]	2	2	2	1	1	0	2	1	2	2	2	2	1	2	2	2	2	2	30	83.3	High
Del Din et al. (2016) [64]	2	2	2	1	1	0	2	1	2	2	2	2	2	1	2	2	2	2	30	83.3	High
Ghasemzadeh and Jafari (2011) [65]	2	1	1	1	0	0	2	2	2	1	0	2	2	1	0	1	2	0	20	55.6	Moderate
Ghobadi and Esfahani (2017) [66]	2	2	1	1	0	0	2	1	2	2	1	2	2	1	2	2	2	1	26	72.2	High
Grimpampi et al. (2016) [67]	2	2	2	1	1	0	2	2	2	2	2	2	2	1	2	2	2	1	30	83.3	High
Kianifar et al. (2017) [68]	2	2	2	2	0	0	2	1	2	2	2	2	2	2	2	2	2	2	31	86.1	High
Lander et al. (2020) [69]	2	2	2	2	2	0	2	2	2	1	2	2	2	2	2	2	2	2	33	91.7	High
Lee et al. (2013) [70]	2	2	2	0	1	0	2	2	2	2	0	2	2	2	2	2	2	2	29	80.6	High
Lin et al. (2021) [71]	1	2	2	2	1	0	2	2	2	2	0	2	2	2	2	2	2	2	30	83.3	High
Liu et al. (2020) [72]	2	2	2	0	0	0	2	1	2	1	0	2	2	2	2	2	2	2	26	72.2	High
Masci et al. (2013) [73]	2	2	2	2	1	0	2	2	2	2	2	2	2	2	2	2	2	0	31	86.1	High
Meng et al. (2019) [74]	2	2	1	0	0	0	2	1	2	1	0	2	2	2	2	2	2	1	24	66.7	Moderate
Michaud et al. (2021) [75]	2	2	2	1	1	0	2	1	2	2	2	2	2	2	2	2	2	2	31	86.1	High
Mitternacht et al. (2022) [76]	2	2	2	1	1	2	2	2	2	2	0	2	2	2	2	2	2	2	32	88.9	High
O’Reilly et al. (2017a) [77]	2	2	2	2	1	0	2	2	2	2	2	2	2	1	2	2	2	2	32	88.9	High
O’Reilly et al. (2017b) [78]	2	2	2	2	1	0	2	2	2	2	2	2	2	1	2	2	2	2	32	88.9	High
Shepherd et al. (2017) [79]	2	2	2	0	0	0	2	1	2	2	2	2	2	2	2	2	2	0	27	75.0	High
Shuai et al. (2022) [80]	2	2	2	2	1	0	2	1	2	2	2	2	2	2	2	2	2	2	32	88.9	High
Spilz & Munz (2022) [81]	2	2	2	1	1	0	2	1	2	2	2	2	2	2	2	2	2	1	30	83.3	High
Tabrizi et al. (2021) [82]	2	2	2	1	1	0	2	1	2	2	2	2	2	1	2	2	2	2	30	83.3	High
Tulipani et al. (2018) [83]	2	1	2	2	1	0	2	1	2	2	2	2	2	2	2	2	2	2	31	86.1	High
Xu et al. (2015) [84]	1	1	1	1	0	0	2	2	2	1	0	2	2	1	2	1	2	1	22	61.1	Moderate
Zhang et al. (2014) [85]	2	0	2	2	0	0	2	0	2	2	2	2	2	2	0	2	2	2	26	72.2	High

Quality assessment criteria: 1. Were the research objectives or aims clearly stated? 2. Was the study design clearly described? 3.Was the study population/sample adequately described? 4. Were the eligibility criteria specified? 5. Was the sampling methodology appropriately described? 6. Was the sample size used justified? 7. Did the method description enable accurate replication of the measurement procedures? 8. Was the assessed movement(s) sufficiently standardised? 9. Was the equipment design and set up clearly described? 10. Were sensor locations accurately and clearly described? 11. Was the sensor attachment method clearly described? 12. Was the signal/data handling described? 13. Were the main outcomes measured and the related calculations (if applicable) clearly described? 14. Was the system compared to an acknowledged gold standard or other acceptable reference standard? 15. Were measures of reliability/accuracy of the equipment used relative to the reference standard system for the intended outcome reported? 16. Were the main findings of the study stated? 17. Were the statistical tests appropriate? 18. Were limitations of the study clearly described? Criteria Scores: 0 = no evidence, 1 = limited evidence, 2 = criteria met

### Device and Sensor Specifications and Applications

An array of devices was utilised in the included studies, with little commonality (Table 4). The sensor configurations and their applications within each study are detailed in Tables 5 and 6, respectively. All included studies utilised a wearable device that included an accelerometer, either in isolation (*n* = 5), in combination with a gyroscope (*n* = 9), or with both a gyroscope and a magnetometer (*n* = 13). Notably, however, eight of the 27 studies did not utilise the full sensory capabilities of the device within their study (Table 4) [62, 67–69, 76, 81, 83, 84]. There was a similar degree of variation in the sampling frequencies utilised (50–200 Hz; Table 4), which were reported in all but one study [59].

**Table 4:** Wearable device systems

Study	Device name	Components				Sampling frequency
		Accelerometer	Gyroscope	Magnetometer	Other	
Ahmadi et al. (2015) [59]	Novel system developed	X	X			–
Beange et al. (2019) [60]	MetaMotionR	X	X	X		100 Hz
Bisi et al. (2017) [61]	Opal	X	X	X		128 Hz
Caporaso and Grazioso (2020) [62]	G-Sensor 2	X	X^a^			200 Hz
Cortesi et al. (2019) [63]	Opal	X	X	X		128 Hz
Del Din et al. (2016) [64]	AX3	X				50/100 Hz^b^
Ghasemzadeh and Jafari (2011) [65]	TelosB	X	X			50 Hz
Ghobadi and Esfahani (2017) [66]	x-IMU	X	X	X		50 Hz
Grimpampi et al. (2016) [67]	Opal	X	X	X^a^		128 Hz
Kianifar et al.(2017) [68]	3-Space (specific model not stated)	X	X	X^a^		90 ± 10 Hz, resampled to 200 Hz
Lander et al. (2020) [69]	MVN Awinda wireless motion capture suit	X	X^a^	X^a^	X^a^	50 Hz
Lee et al. (2013) [70]	Derivative of an inertial measurement system developed by [90]	X				100 Hz
Lin et al. (2021) [71]	Novel system developed	X	X	X		75 Hz
Liu et al. (2020) [72]	Novel inertial measurement system developed by Dalian University of Technology	X	X	X		148 Hz
Masci et al. (2013) [73]	FreeSense	X	X			100 Hz
Meng et al. (2019) [74]	Trigno (specific model not stated)	X	X			51.2 Hz
Michaud et al. (2021) [75]	STT-IWS	X	X	X		100 Hz
Mitternacht et al. (2022) [76]	InvenSense ICM-20948	X	X	X^a^		200 Hz
O’Reilly et al. (2017a) [77]	Shimmer3 IMU	X	X	X		51.2 Hz
O’Reilly et al. (2017b) [78]	Shimmer3 IMU	X	X	X		51.2 Hz
Shepherd et al. (2017) [79]	SABELSense	X	X	X		100 Hz
Shuai et al. (2022) [80]	Perception Neuron	X	X	X		100 Hz
Spilz & Munz (2022) [81]	Shimmer3 IMU	X	X	X	X^a^	120 Hz
Tabrizi et al. (2021) [82]	BNO055	X	X	X		70 Hz
Tulipani et al. (2018) [83]	Opal	X	X	X^a^		128 Hz
Xu et al. (2015) [84]	InvenSense MotionFit	X	X^a^	X^a^		200 Hz
Zhang et al. (2014) [85]	TEMPO 3.1 [91]	X	X			100 Hz

^a^Sensor was present but not utilised as part of the study

^b^50 Hz initially, then device upgraded during the study to accommodate 100 Hz, which was resampled to 50 Hz

**Table 5:** Included studies using a classification method to assess movement quality

Study	Participants	Utilised sensors and placements	No. devices	Application	Categorisation	Classification method, input variables and features	Validation method	Findings
Bisi et al. (2017) [61]	45 healthy children (28 boys; 6–10 years)	Tri-axial accel + gyro + mag (low back, ankle L&R, wrist L&R)	5	Motor competence of FMS	Sequential binary technique classifiers (3–4 criteria per movement)	Feature-based classification Signal features extracted from raw accel, gyro, and mag time-series data	Real-world validation by three experts analysing video footage	Performance criteria classification accuracy = 77–100% Overall skill classification accuracy = 87–96%
Caporaso and Grazioso (2020) [62]	9 healthy adults (7 male; 25.3 ± 4.7 years)	Tri-axial accel (low back)	1	Race walking infringement detection	Binary infringement classification Three-level infringement classification Fuzzy classification	Feature-based classification Signal features extracted from raw accel time-series data	Real-world validation using video footage from a high-speed camera	Binary infringement classification accuracy = 87% Three-level infringement classification accuracy = 63% Fuzzy classification accuracy = 92%
Ghasemzadeh and Jafari (2011) [65]	3 healthy adults (3 males; 25–35 years)	Tri-axial accel + bi-axial gyro (chest, hip, wrist)	3	Coordination analysis of a baseball swing	Transcript-based coordination analysis to distinguish between proper and improper technique	Statistical feature-based classification utilising k-means clustering Signal features extracted from raw accel and gyro time-series data	Real-world validation using video footage from two webcams which was synchronised with the sensors	The overall error of the sensor-based approach over all categories was 101 ms, equal to 3.4% of the total length of the template (3 s) when compared with the video-based analysis
Ghobadi and Esfahani (2017) [66]	Part 1: 10 healthy adults (sex and age not specified) Part 2: 2 healthy adults from Part 1 sample (1 male; age not specified)	Tri-axial accel + gyro + mag (ankle)	1	Gait abnormality monitoring	Multi-label activity classification	SVM Signal features extracted from raw accel, gyro, and mag time-series data and from orientations obtained through Kalman-based sensor fusion	KF-CV	Detection of abnormal walking accuracy = 99%
Grimpampi et al. (2016) [67]	58 healthy children (30 boys; 8 ± 2 years)	Tri-axial accel + gyro (low back, chest, wrist)	3	Motor competence of overarm throwing	Multi-label development level classification	Feature-based classification Signal features extracted from raw accel and gyro time-series data, and derived kinematic parameters	Real-world validation by one expert analysing video footage (camera details not provided)	Between three development levels (DLTC), a significant difference (*p* = 0.001) was achieved between DLTC_1_ and DLTC_3_, and DLTC_2_ and DLTC_3_, using temporal parameters for the cocking phase of an overarm throw. No significant difference was observed using other temporal parameters. Trunk max acc_AP_ in DLTC_3_ was significantly higher (*p* < 0.0005) than in DLTC_1_ and DLTC_2_, with no significant difference between DLTC_1_ and DLTC_2_. A significant difference (*p* < 0.0005) between DLTC_1_ and DLTC_2_ for ROM_Pitch_ was also revealed
Kianifar et al.(2017) [68]	14 healthy adults (7 males; 30.8 ± 5.5 years)	Tri-axial accel + gyro (low back, thigh, shank)	1 and 3	Technical performance the single-leg squat in a clinical setting	Binary technique classification Three-level technique classification	SVM KNN NB Signal features extracted from raw accel, gyro, and mag time-series data and from joint angles obtained through Kalman-based sensor fusion	KF-CV LOSOCV	Binary classification (KF-CV): 3 IMU–accuracy = 96%, single IMU (ankle)–accuracy: 90% Three-level classification (KF-CV): 3 IMU–accuracy = 65%, single IMU (ankle)–accuracy: 64% Binary classification (LOSOCV): 3 IMU–accuracy = 90%, single IMU (ankle)–accuracy: 69% Three-level classification (LOSOCV-CV): 3 IMU–accuracy = 60%, single IMU (ankle)–accuracy: 50%
Lander et al. (2020) [69]	14 healthy children (9 boys; 7–12 years)	Tri-axial accel (ankle L&R, wrist L&R)	4	Motor competence of FMS	Sequential binary technique classifiers (3–4 criteria per movement)	k-means clustering Signal features extracted from raw accel time- and frequency-domain data	Real-world validation by two experts analysing video footage and data from an IMU motion capture suit	Performance criteria classification accuracy = 72–100% Overall skill classification accuracy = 77–100%
Lee et al. (2013) [70]	7 healthy adults (5 males; 25 ± 7.3 years)	Tri-axial accel (low back)	1	Race walking infringement detection	Binary infringement classification	Feature-based classification Signal features extracted from raw accel time-series data	Real-world validation using video footage	Binary infringement classification accuracy = 91%
Liu et al. (2020) [72]	6 (sex, age and medical status not specified)	Tri-axial accel + gyro + mag (upper arm L&R, forearm L&R, low back, mid back, thigh L&R, shank L&R, foot L&R)	12	Canoeing proficiency level	Binary proficiency level classification	SVM Logistic regression Decision tree XGBoost Signal features extracted from raw accel, gyro, and mag time- and frequency-domain data and from joint angles obtained through Madgwick [48] sensor fusion	KF-CV	SVM general features accuracy = 100%, selected features accuracy = 97% Logistic regression general features accuracy = 99%, selected features accuracy = 96% Decision tree general features accuracy = 94%, selected features accuracy = 94% XGBoost general features accuracy = 100%, selected features accuracy = 99%
Masci et al. (2013) [73]	54 healthy children (sex not specified; 5 ± 3 years)	Tri-axial accel + gyro (low back)	1	Motor competence of running based on arm actions	Multi-label technique classification	Feature-based classification Signal features extracted from raw accel and gyro time- and frequency-domain data, and derived kinematic parameters	LOOCV	DLAA detection accuracy = 67–87%
O’Reilly et al. (2017a) [77]	80 healthy adults (57 males; 24.68 ± 4.91 years)	Tri-axial accel + gyro + mag (low back, thigh L&R, shank L&R)	1–5	Technical performance of a barbell deadlift	Binary technique classification Multi-label technique classification	Random forests Signal features extracted from raw accel, gyro, and mag time- and frequency-domain data and from joint angles obtained through Madgwick [48] sensor fusion	Global: LOSOCV Personalised LOOCV	Binary classification with natural deviations using a global model (LOSOCV): 5 IMU–accuracy = 73%, single IMU (right thigh)–accuracy: 71% Binary classification with natural deviations using personalised model (LOOCV): 5 IMU–accuracy = 84%, single IMU (right thigh)–accuracy = 82% Multi-class classification of natural deviations using global model (LOSOCV): 5 IMU–accuracy = 54%, single IMU (right shank)–accuracy = 53% Multi-class classification of natural deviations using personalised model (LOOCV): 5 IMU–accuracy = 78%, lumbar IMU–accuracy = 75%
O’Reilly et al. (2017b) [78]	77 healthy adults (55 males; 22.63 ± 4.87 years)	Tri-axial accel + gyro + mag (low back, thigh L&R, shank L&R)	1–5	Technical performance of a bodyweight squat	Binary technique classification Multi-label technique classification	Random forests Signal features extracted from raw accel, gyro, and mag time- and frequency-domain data and from joint angles obtained through Madgwick [48] sensor fusion	LOSOCV	Binary classification (LOSOCV): 5 IMU–accuracy = 98%, single IMU (left thigh)–accuracy: 98% Multi-class classification (LOSOCV): 5 IMU–accuracy = 80%, single IMU (right shank)–accuracy = 73%
Spilz & Munz (2022) [81]	17 healthy adults (9 males; 24–62 years)	Tri-axial accel + gyro + mag (forehead, chest, upper arm L&R, forearm L&R, hand L&R, mid back [scapulae] L&R, low back, thigh L&R, shank L&R, foot L&R)	17	Technical performance of Functional Movement Screen [92] exercises	Three-level proficiency classification	CNN-LSTM layered neural network Features determined by CNN layer of the neural network	KF-CV LOSOCV	Mean of the macro F1-scores [93] for known participants: Training data = 0.58–0.95, validation data = 0.57–0.95, test data = 0.55–0.91 Mean weighted F1-scores [93] for unknown participants: Training data = 0.81–0.98, validation data = 0.79–0.95, test data = 0.15–0.49
Tabrizi et al. (2021) [82]	16 healthy adults (mixed sex; 19–38 years)	Tri-axial accel + gyro + mag (racket blade centre)	1	Technical performance of table tennis forehand strokes	Multivariate regressiona	SVR LSTM CNN Signal features extracted from raw accel, gyro, and mag time-series data and from orientations obtained through Madgwick [48] sensor fusion	KF-CV	SVR: Weighted average of ${\overline{R} }^{2}$ = 0.962 LSTM: Weighted average of ${\overline{R} }^{2}$ = 0.997 CNN: Weighted average of ${\overline{R} }^{2}$ = 0.958 The weighted average of RMSE showed the LSTM models to have lower estimation error than SVR (4.24%) and CNN (33.79%)
Xu et al. (2015) [84]	6 healthy adults (4 males; age not specified)	Tri-axial accel (foot)	1	Cycling pedal profiling	Multi-label technique classification	SVM Signal features extracted from raw accel time-series data	KF-CV	Pedal profile classification accuracy = 84–99%
Zhang et al. (2014) [85]	17 healthy adults (9 males; 29 ± 11 years)	Tri-axial accel + bi-axial gyro (*x* nd y axes) + uni-axial gyro (*z* axis) (shank, chest)	2	Gait abnormality monitoring	Binary fatigue status classification	SVM Signal features extracted from raw accel and gyro time- and frequency-domain data, and derived kinematic parameters	KF-CV	Intra-individual general features accuracy = 97% Inter-individual general features accuracy = 90% Inter-individual selected features accuracy = 93% Inter-individual selected features accuracy = 88%

Findings are reported to the degree of accuracy provided in the original study

*accel* accelerometer, *CNN* convolutional neural network, *DLAA* development level (arm action), *DLTC* development level (trunk component), *FMS* fundamental movement skill, *gyro* gyroscope, *IMU* inertial measurement unit, *KF-CF* k-fold cross-validation, *KNN* k nearest neighbourhood, *L* left, *LOOCV* leave-one-out cross-validation, *LOSOCV* leave-one-subject-out cross-validation, *LSTM* long short-term memory, *mag* magnetometer, *NB* naïve Bayes, *R* right, ${\overline{R} }^{2}$ adjusted coefficient of determination, *ROM*_*Pitch*_ range of motion of pitch, *SVM* support vector machine, *SVR* support vector regression, *Trunk max acc*_AP_ maximum antero-posterior linear acceleration

^a^By definition, multivariate regression modelling did not feature distinct classes; rather, regression algorithms were utilised for problems featuring continuous and numeric outputs [94]

**Table 6:** Studies using motion-based measurements to assess movement quality

Study	Participants	Sensors and placements	Qty	Application	Measurement method	Gold/reference standard	Findings
Ahmadi et al. (2015) [59]	10 adults (9 healthy, 1 with low back pain; sex and age not specified)	Tri-axial accel + gyro (shank L&R, thigh L&R,	6	Joint angle measurement when jogging	Sensor orientations calculated using the Madgwick algorithm [48] with joint angles determined based on sensor orientations on proximal and distal segments	Joint angle measurements generated using normative data and applying a phase shift registration algorithm	The unregistered mean knee angle curve demonstrated significantly higher (*p* = 0.002) magnitudes for 11%–17% and 87%–96% of the foot contact cycle and lower magnitudes (*p* < 0.001), respectively, and for 61%–75% of the foot contact cycle. The unregistered mean hip angle curve had significantly lower (*p* < 0.010) magnitudes for 14–22% and 74–86% of the foot contact cycle
Beange et al. (2019) [60]	10 healthy adults (6 males; males: 25.3 ± 2.2 years, females: 22.8 ± 2.2 years)	Tri-axial accel + gyro + mag (mid back, low back)	2	Measurement of lumbar spine flexion and extension	Sensor orientations calculated using a Kalman filter with flexion–extension angles obtained based on orientations of each sensor relative to one another	10-camera optical motion capture system (Vicon VantageV5, Vicon Motion Systems Ltd., Oxford, UK)	ICC when using both the FE angle time-series, and the SS time-series using the FE angle, lateral bend angle, and axial twist angle, to measure local dynamic stability (0.807 ≤ ICC_FE_ ≤ 0.919; 0.738 ≤ ICC_SS_ ≤ 0.868) ICC for sagittal plane lumbopelvic coordination (0.961 ≤ ICC_MARP_ ≤ 0.963), ICC for sagittal plane lumbopelvic variability (0.961 ≤ ICC_DP_ ≤ 0.963)
Cortesi et al. (2019) [63]	14 healthy adults (14 males; 23.2 ± 2.8 years)^a^	Tri-axial accel + gyro + mag (chest, upper arm L&R, forearm L&R)	5^b^	Measurement of swimming stroke wrist trajectory	Wrist trajectories were determined using the Madgwick algorithm [48] coupled with the kinematic chain model [95]	7-camera optical motion capture system (BTS SMART-DX 7000, BTS Bioengineering, Milan, Italy)	RMSE between optical motion capture and sensor wrist trajectory = 7.7 cm 3D mean distance between optical motion capture wrist trajectory and sensor wrist trajectory = 13.0 ± 4.5 cm Correlation (r) between optical motion capture and the wearable sensors = 0.94
Del Din et al. (2016) [64]	60 adults (30 healthy, 30 with PD; sex not specified; healthy: 66.6 ± 7.7 years, PD: 66.9 ± 9.4 years)	Tri-axial accel (low back)	1	Measurement of gait step length	Step length measured using the inverted pendulum model [89]	7.0-m long × 0.6-m wide instrumented walkway (Platinum model GaitRite, software version4.5, CIR systems, NJ, USA) and a synchronised webcam (Logitech, Webcam Pro 9000, CA, USA)	ICC between measurement accelerometer and instrumented walkway was 0.913 for healthy participants and 0.869 for those with PD
Lin et al. (2021) [71]	13 adults (10 healthy, 8 males; 3 with FS, sex not specified; 23.3 ± 1.3 years)	Tri-axial accel + gyro + mag (upper arm L, forearm L, chest)	3	Measurement of shoulder ROM	Shoulder ROM calculated using a complementary filter to determine rotation angles	Optical motion capture system (Vicon Motion Systems Ltd. Oxford, UK)	Mean RMSE for all ROM tests ranged from 2.5° to 3.6°
Liu et al. (2020) [72]	6 (sex, age and medical status not provided)	Tri-axial accel + gyro + mag (upper arm L&R, forearm L&R, low back, mid back, thigh L&R, shank L&R, foot L&R)	12^c^	Joint angle measurement during a canoeing stroke	Sensor orientations calculated using the Madgwick [48] algorithm with joint angles determined based on sensor orientations on proximal and distal segments	12-camera optical motion capture system (OptiTrack, Natural Point Inc. USA)	Mean error for joint angles ranged from 1.20 to 3.72%
Meng et al. (2019) [74]	10 healthy adults (6 males; 26.5 ± 6.2 years)	Tri-axial accel + gyro (shank, foot)	2	Measurement of ankle angle when walking	Orientation of each sensor is calculated using a complementary filter where the ankle joint angles are subsequently calculated	12-camera optical motion capture system (Vicon MX Giganet, Vicon Motion Systems Ltd. Oxford, UK)	RMSE of the sensors compared to the optical motion capture system was < 3.5° for all measured walking speeds
Michaud et al. (2021) [75]	39 healthy adults (21 males; 39 ± 21 years)	Tri-axial accel + gyro + mag (low back, mid back)	2	Measurement of lumbar flexion and pelvic tilt when performing hip hinge exercises	Sensor orientations calculated using the Madgwick [48] algorithm with lumbar flexion angles estimated based on sensor orientations	8-camera optical motion capture system (OptiTrack FLEX 3, Natural Point Inc. USA)	Lumbar flexion (2 IMUs): RMSE of 2.9° and 2.5° throughout movement and 2.7° and 3.0° at maximum angles for American kettlebell swing and deadlift, respectively Pelvic tilt (1 IMU): RMSE of 2.4° and 1.9° at maximum angles for American kettlebell swing and deadlift, respectively
Mitternacht et al. (2022) [76]	5 healthy adults (5 males; 25–61 years)	Tri-axial accel + gyro (shank)	1	Measurement of motor control during a battery of functional movement tests^d^	Kinematic variables obtained through integration of short sections of IMU time series data extracted from longer analysis sequences	8-camera optical motion capture system (Simi Reality Motion Systems GmbH, Unterschleissheim, Germany)	One-leg squat test for knee valgus: RMSE = 1.4° Drop jump squat test for knee valgus: RMSE = 2.2°, ΔΧ was 47% lower for the IMU (8.6 mm) than the camera system (16.2 mm)
Shepherd et al. (2017) [79]	10 healthy adults (0 males)	Tri-axial accel + gyro + mag (forearm)	1	Measurement of forearm angle during a netball shot	Madgwick [48] algorithm used to determine the pitch angle of the wearable device relative to the Earth’s magnetic frame of reference from which the forearm angle was derived	10-camera optical motion capture system (Vicon VantageV16, Vicon Motion Systems Ltd. Oxford, UK)	The sensors were found to overestimate the forearm angle by 4.03 ± 1.58% on average across all shots compared to the optical motion capture system
Shuai et al. (2022) [80]	20 healthy adults (10 males; 25.19 ± 2.8 years)	Tri-axial accel + gyro + mag (low back, thigh L&R, shank L&R, foot L&R)	7	Measurement of hip, knee, and ankle angles during a battery of functional tests	Sensor orientations calculated using a Kalman filter with joint angles obtained based on orientations of each sensor relative to one another	8-camera optical motion capture system (OptiTrack Prime17W, Natural Point Inc. USA)	Ankle RMSE before zero offsetting: hip = 4.7°–9.3°, knee = 3.6°–11.0°, ankle = 4.4°–13.1° Ankle RMSE after zero offsetting: hip = 3.0°–7.5°, knee = 2.1°–10.0°, ankle = 3.4°–12.8°
Tulipani et al. (2018) [83]	10 healthy adults (5 males)	Tri-axial accel + gyro (low back, pelvis, thigh L&R, shank L&R)^e^	6	Measurement of angular displacement of body segments during functional tasks	Angular velocity data from gyroscopes were integrated to obtain angular displacement using an undisclosed algorithm	9-camera optical motion capture system (Qualisys AB, Gothenburg, Sweden)	Overall average RMSE of the sensors compared to the optical motion capture system for the for angular displacement across all tasks and segments was 3.7° (RMSE range = 2.0°–7.1°)

Findings are reported to the degree of accuracy provided in the original study

*3D* three-dimensional, *accel* accelerometer, *DP* deviation phase, *FE* flexion–extension, *FS* frozen shoulder, *gyro* gyroscope, *ICC* intra-class correlation coefficient, *L* left, *mag* magnetometer, *MARP* mean absolute relative phase, *PD* Parkinson’s disease, *R* right, *r* correlation, *RMSE* root-mean-square error, *ROM* range of motion, *SS* sum of squares, *ΔΧ* mean medial shift

^a^The phase of study that was applicable for the systematic review only featured five participants from the total sample

^b^While five sensors were worn in the study, only one device was worn when measuring the wrist trajectory

^c^The authors suggested that not all sensors were used in the measurement of joint angles in this study, stating that only sensors on the upper limbs were considered. However, it is not clear which of the sensors were actually used

^d^While the study featured a battery of 10 movement tests, only two movements met the inclusion criteria for this systematic review: one-leg squat and drop jump

^e^Only angular velocity data were presented in the paper, suggesting the use of the gyroscope exclusively. However, the accelerometer may have been used in the calibration and cannot be omitted without further information

The wearable devices were positioned across a broad range of anatomical locations (Tables 5 and 6), most commonly on the lower back (*n* = 15; [60–62, 64, 67, 68, 70, 72, 73, 75, 77, 78, 80, 81, 83]) and distal leg segments, encompassing the shanks and ankles (*n* = 14; [59, 61, 66, 68, 69, 72, 74, 76–78, 81, 83–85]). Other anatomical locations for the sensors were the wrists or forearms (*n* = 8; [61, 63, 67, 69, 71, 72, 79, 81]), thighs (*n* = 8; [59, 68, 72, 77, 78, 80, 81, 83]), chest (n = 6; [63, 65, 67, 71, 81, 85]), feet (*n* = 5; [72, 74, 80, 81, 84]), mid back (*n* = 4; [60, 72, 75, 81]), upper arms (*n* = 4; [63, 71, 72, 81]), pelvis (*n* = 1; [65]), hip (*n* = 1; [83]), hand (*n* = 1; [81]), and head (*n* = 1; [81]). Additionally, one study positioned a single wearable device on a piece of sports equipment, specifically a table tennis racket [82]. The number of wearable devices used ranged from one to 17, with 25 of the 27 studies featuring seven or fewer devices. Moreover, it was indicated by Liu et al. [72] that not all 12 devices were utilised throughout their entire study; only the upper body was considered in the measurement of joint angles.

Sensor fusion was a common feature across the included studies, with 14 of the 27 studies capitalising on such an approach. The Madgwick algorithm [48] was employed in seven studies [59, 63, 72, 75, 77–79], while a Kalman filter [86] was used in five studies [60, 66, 68, 80, 82], and complementary filters [87, 88] were used in two studies [71, 74]. When using wearables to measure kinematics, there were exceptions to the use of sensor fusion, with Del Din et al. [64] utilising a single accelerometer, Mitternacht et al. [76] using both accelerometers and gyroscopes, and Tulipani et al. [83] employing gyroscopes exclusively. In the studies by Del Din et al. [64] and Tulipani et al. [83], sensor fusion was not possible due to the omission of other sensors. Rather, Del Din et al. [64] applied the inverted pendulum model [89] to measure step length, while Tulipani et al. [83] integrated the angular velocity obtained from the gyroscope using a proprietary algorithm to calculate the angular displacement of body segments. Mitternacht et al. [76] also opted to utilise an integration method for the movements considered within this systematic review, integrating the angular velocity to determine angles, and double integrating acceleration to calculate linear position. The influence of integration drift [32] was reduced by dividing longer time series data into shorter segments [76]. Where sensor fusion was used to classify movement characteristics [66, 68, 72, 77, 78, 82], the orientation data were utilised to expand the available range of features for implementation within the classification algorithms.

### Descriptive Aspects of Reviewed Studies

Of the 27 studies included in the systematic review, 16 featured methods that were used to classify movement qualities or abnormalities (Table 5), and 12 used methods to measure motion-based characteristics (Table 6) to assess movement quality, typically through the measurement of joint angles or segment-rotation angles [59, 60, 63, 71, 72, 74–76, 79–81, 83]. Notably, one study directly measured motion to assess movement quality while also employing classification methods, and was therefore included in both study groupings [72]. Four of the 27 studies used device-based measures to assess the movement of healthy children using wearables [61, 67, 69, 73], all of which utilised classification methods for assessing motor competence when performing fundamental movement skills (FMS). Of the remaining studies, 19 featured healthy adult samples [60, 62, 63, 65, 66, 68, 70, 74–85], three included both healthy participants and participants with a pathology for comparative purposes [59, 64, 71], while one study did not report participant details [72]. Additionally, 11 of the 27 studies were conducted in a clinical setting for medical applications [59, 60, 64, 66, 68, 71, 74, 85], with the remainder focused on sports performance or sports-injury prevention; all included articles were feasibility studies. Sample sizes ranged from two to 77 participants, though 20 studies featured small sample sizes of 20 or fewer participants [59, 60, 62, 63, 65, 66, 68–72, 74, 76, 79–85]. 

### Classification and Validation Methods Used to Assess Movement Quality

Ten of the 16 classification studies incorporated a binary-classification approach based on the data obtained from the wearable sensors [61, 62, 65, 68–70, 72, 77, 78, 85], while only nine studies applied a multi-class classification [62, 66–68, 73, 77, 78, 84]. Four studies employed and compared both binary- and multi-class methods [62, 68, 77, 78]. In addition, Tabrizi et al. [82] employed multivariate regression models, which, by definition, use continuous and numeric outputs rather than featuring distinct classes. Given the comparable characteristics between classification and regression models, they have been categorised together for this review. Specifically, Tabrizi et al. [82] used conventional machine-learning methods, namely support vector regression (SVR), and two deep-learning methods, convolutional neural networks (CNN) and long short-term memory (LSTM), which were used to generate movement quality scores using set criteria [82]. For the true classification studies, where accuracies were presented as percentages, binary skill-classification accuracy ranged from 69 to 100% (Table 5) [61, 62, 68–70, 72, 77, 78, 85]. Ghasemzadeh and Jafari [65] also applied a binary-classification approach, though the results were defined in terms of the percentage error of the wearable system relative to analysed video footage (3.4%). Furthermore, while Ghobadi and Esfahani [66] utilised a multi-class approach to distinguish different locomotor activities, there appears to be an intra-classification binary approach used to distinguish healthy and erroneous gait patterns, with 99% accuracy reported for the detection of abnormalities. Where a multi-class approach was otherwise used, accuracies ranged from 53 to 99% [62, 68, 73, 77, 78, 84]. Grimpampi et al. [67], and Spilz and Muntz [81] also used multi-class classification approaches, though the findings were reported based on the significance of differences between signal features in the former, and in terms of F1-scores [93] in the latter.

A range of classification methods were applied within the included studies (see Table 5). Most common were feature-based classification methods, an overarching term used to encompass traditional feature-learning methods, utilised in five of the 16 studies [61, 62, 67, 70, 73], and SVM, also used in five studies [66, 68, 72, 84, 85]. SVR, derived from traditional SVM modelling, was also applied in Qiu et al. [82]. Other classifiers used throughout the included studies were k-nearest neighbour (KNN) [68], naïve Bayes (NB) [68], k-means clustering [65, 69], logistic regression [72], decision tree [72], XGBoost [72], random forests [77, 78], CNN [81, 82], and LSTM [81, 82]. Three studies employed multiple classifiers to determine the highest-performing methods for their respective applications [68, 72, 82]. Across the range of classifiers, the studies employed different approaches to feature selection (Table 5), although, with the exception of Spilz and Munz [81], the studies typically utilised raw sensor time-series data (*n* = 15). However, it was also common to derive kinematic data from the raw data (*n* = 3) [67, 73, 85] and capitalise on sensor fusion (*n* = 6) [66, 68, 72, 77, 78, 82] to expand the available features, while also extracting features from the raw data in the frequency domain (*n* = 6) [66, 68, 72, 77, 78, 82]. An assortment of validation methods was applied to the classifiers throughout all included studies; six studies used a real-world validation approach based on video footage [61, 62, 65, 67, 69, 70]. However, statistical methods were typically preferred, with seven studies using a k-fold cross-validation (KF-CV) [66, 68, 72, 81, 82, 84, 85], four a leave-one-subject-out cross-validation (LOSOCV) [68, 77, 78, 81], and two a leave-one-out cross-validation (LOOCV) [73, 77].

### Methods and Reference Standards for the Measurement of Kinematic Characteristics to Assess Movement Quality

Sensor-fusion algorithms enable the calculation of sensor orientation, providing details of angular rotation about each of the three axes when considering movement in three-dimensional space. When wearable devices are positioned either side of a joint, or joints, the orientations relative to one another can enable joint angles to be estimated. A sensor-fusion approach was taken in 9 of the 12 measurement studies, including Cortesi et al. [63], where the kinematic chain model was used in conjunction with sensor fusion to generate both rotational and translational information [95]. Shepherd et al. [79] used sensor fusion in a simpler concept by calculating forearm angles using the orientation of a single device relative to the ground, comparable with Lin et al. [71], who utilised certain components of a shoulder range of motion (ROM) assessment. Similarly, Tulipani et al. [83], using gyroscope data exclusively, determined angular displacement for body segments rather than joint angles, a method also adopted by Mitternacht et al. [76], who measured tibial tilt angles for assessing knee valgus. However, Mitternacht et al. [76] also identified the linear medial shift of the knee joint by double-integrating the mediolateral acceleration obtained from the accelerometer. Del Din et al. [64] was the only study to exclusively measure translational movement without rotation or orientation, also using an integration-based method to obtain displacement and thereby calculate the length of a step.

To validate measurements of motion using novel instrumentation or methods, a predetermined reference standard is typically utilised for comparison, as is the case for all 12 of the measurement-based studies included in this review (see Table 6). Del Din et al. [64], for example, used an instrumented walkway to validate the measurement accelerometer, providing a strong intra-class correlation coefficient (ICC) for both healthy participants (ICC = 0.913) and for those with Parkinson’s disease (ICC = 0.869). However, optical motion capture, generally considered to be the gold-standard for measuring movement [22], was utilised by 10 of the 12 measurement studies to validate the wearable sensor measurements (Table 6) [60, 63, 71, 72, 74–76, 79, 80, 83]. Six studies evaluated the accuracies of the angles calculated using the wearable sensor data in terms of root-mean-square error (RMSE) when compared to the optical motion capture systems [71, 74, 75, 76, 80, 83]. Considering three different walking speeds (0.5, 1.0 and 1.5 m‧s^−1^), Meng et al. [74] reported that the RMSE was always less than 3.5°. This is congruent with Tulipani et al. [83], where the overall average RMSE across a series of movements was 3.7°, and Lin et al. [71], who observed a maximum mean RMSE of 3.6° across the series of tests conducted. Moreover, Michaud et al. [75] found similar results when monitoring lumbar flexion and pelvic tilt, with RMSE ranging from 1.9° to 3.0° for the barbell deadlift and the American kettlebell swing. Mitternacht et al. [76] also reported comparable results, where the RMSE ranged from 1.4° to 2.2°. However, whilst the measurements were captured to determine knee instability during movements featuring knee flexion, namely the one-leg squat and drop jump, knee instability was not evident, resulting in minimal movement of the IMU. Consequently, the observed measurement errors were small [76]. Shuai et al. [80] reported a greater degree of error, with RMSE ranging from 2.1° to 13.1° across all measured joints and configurations. Cortesi et al. [63] evaluated the translational motion of a wrist-worn device against an optical motion capture system and reported a RMSE of 7.70 cm. Both Shepherd et al. [79] and Liu et al. [72] stated the mean error as percentages, indicating that the sensors overestimated the forearm angle by an average of 4.03% and calculating a maximum mean error of 3.72%, respectively. Beange et al. [60] reported on the reliability of wearable sensors when compared against an optical motion capture system for measuring spinal motion. Specifically, the authors reported on spinal flexion–extension angles (0.807 ≤ ICC_FE_ ≤ 0.919), and the sum of squares of flexion–extension, lateral bend, and axial twist angles to measure local dynamic stability (0.738 ≤ ICC_SS_ ≤ 0.868) [60]. Ahmadi et al. [59] utilised normative data and applied a phase-shift registration algorithm [96] to refine the joint angle measurements before conducting an intra-study comparison against an unregistered approach. The phase shift, a pre-analysis curve transformation technique that uses a timescale shift based on the position of signal features [96], was used to align flexion–extension curves for foot contact cycles during jogging. Both the registered and unregistered curves were stated by Ahmadi et al. [59] to be representative of the mean joint angles obtained across the sample, where it was observed that the unregistered curve significantly underestimated the joint angle maxima (p = 0.002) and minima (p < 0.001) relative to the phase-shift registration algorithm. While significant, with the authors noting that even small differences could be indicative of injury, or increased risk of injury, they also acknowledged observed similarities between the joint angle curves generated throughout the duration of each movement.

## Discussion

This systematic review sought to highlight any additional benefits that could be gained using multi-sensor devices, or multiple wearable devices, in place of unimodal sensors or a single device, respectively, when assessing movement quality. Accordingly, this review also investigated the additional variables, and indeed features, that could be obtained through multi-sensor devices for use in movement analyses. Further, the systematic review aimed to differentiate between the processing methods and applications of multi-sensor wearable devices in comparison to unimodal sensors when assessing movement quality. Evidence from the current review suggests that most movement quality assessments utilising wearable technology centre around expert-led or expert-based assessments [61, 62, 67–70, 73, 76–81, 83], often capitalising on pre-validated movement screening methods [61, 67, 69, 73, 76, 81]. Alternatively, movements are commonly assessed using proficient performers as the baseline [59, 60, 64, 66, 71, 72, 75, 84, 85]. Nevertheless, there are additional methodological considerations for the assessment of movement quality, and the authors of future reviews are recommended to further consider the specifics surrounding movement-quality assessment methods and the integration of technology. The reviewed studies revealed two overarching themes in the use of wearable technology to assess movement quality, with technology either used to classify movements or to directly measure motion for comparison against a baseline. To distinguish between the two methods, the results of these studies were reported independently, though Liu et al. [72] featured in both measurement and classification categories. It is pertinent to note that accuracies reported for each included study may not be comparable if applied to other movements with alternative sensor placements.

All studies included in the review utilised an accelerometer, a gyroscope, or a magnetometer, or a combination thereof. Accelerometers were the most common of the three sensors, likely due to the versatility and historical application of these sensors when measuring movement [97]. Most often, the sensors were tri-axial and could therefore capture data in three dimensions, which is desirable given that typical human movements are not linearly constrained. Nonetheless, Ghasemzadeh and Jafari [65] used a tri-axial accelerometer coupled with a bi-axial gyroscope, although the two-dimensional angular velocity data obtained from the gyroscope were most likely sufficient for the intended application, as the focus was solely on rotation in the transverse plane.

Across all studies, the sampling frequency did not vary substantially when using a classification or measurement method. The comparable sampling frequencies are possibly related to the type of movements assessed, all of which were gross motor skills. Additionally, the range of sampling frequencies follows existing trends in other areas of the literature for similar applications [21]. Higher sampling frequencies may increase the clarity of motion measurements, particularly in explosive or finer movement patterns [40], although no included study considered fine motor skills and only five of the included studies could be considered to have used explosive movements: a baseball bat swing [65], overarm throw [67], table tennis forehand strikes [82], drop jump [76], and countermovement jump [80]. These studies featured sampling frequencies of 50 Hz, 128 Hz, 70 Hz, 200 Hz, and 100 Hz, respectively, which are relatively low in contrast to the frequencies recommended by Worsey et al. [40], and mostly at the lower end of the range of sampling frequencies observed in this review. Two of these studies [65, 67] successfully utilised a true classification method, suggesting that, for each classification method applied, the sampling frequencies were adequate. Tabrizi et al. [82] used a regression method, which was also successfully implemented using a sampling frequency below the average across this review. While Mitternacht et al. [76] and Shuai et al. [80] both aimed to measure motion during explosive movements, each study has limitations. Specifically, none of the participants in the study by Mitternacht et al. [76] exhibited noticeable knee instability during the drop jump, suggesting that observed tibial movement was minimal. Shuai et al. [80] used a height-restricted countermovement jump to prevent marker occlusion as well as timing controls to reduce the influence of movement speeds on measurements. Consequently, it was not possible based on the evidence provided by the studies included in this review to deduce whether an increased sampling frequency would have an inherent benefit for measuring motion in studies assessing explosive movements, though speculatively, high sampling frequencies may be required to obtain accurate instantaneous measurements for high-speed movements due to the risk of aliasing error [98]. Indeed, it is not uncommon for average measurements to be utilised when measuring fast movements using wearables [99] due to the inability of low-sampling-frequency devices to capture sufficient data during rapid changes in the signal [98]. Yet, what we can determine from this review is that it is feasible to assess movement quality, including rapid human movements, using comparatively lower sampling frequencies when employing classification methods, for which high signal resolution may not be required. Of note, sensors with enhanced design features typically have higher unit costs [100], meaning most cost-effective, commercially available sensors likely avoid particularly high sampling frequencies.

The included studies featuring a classification approach highlighted the variety of factors that can influence the accuracy of classifiers. O’Reilly et al. [77, 78], for example, drew intra-study comparisons, where it was observed that, overall, a decrease in the quantity of sensors used tended to reduce classification accuracy. However, it was reported in both studies that the accuracy was not always markedly diminished by reducing the number of sensors utilised, depending on sensor location. Specifically, it was shown that single sensors worn on the shanks during bodyweight squats [78], or on the lower back or thighs for deadlifts [77] could provide comparable classification accuracies to a configuration with as many as five sensors. However, classification accuracy typically reduced considerably when using fewer sensors in sub-optimal positions for the respective movements, such as the shanks for the deadlift [77] and the lower back for bodyweight squats [78]. This is further evidenced when comparing the results of the two O’Reilly et al. [77, 78] studies, with the multi-class classifier employed for assessing squat technique [78] being noticeably more accurate than the equivalent classifier for the deadlift [77], despite the same sensor placements being used. It is speculated that this is due to the more proximal positions of the sensors to the main area of the observed deviations (i.e., the lower limbs) during the squat, whereas the deviations identified for the deadlift largely overlooked lower limb movements and positions. However, it may be feasible to obtain good classification accuracies with multi-class classifiers using fewer sensors. As indicated by Masci et al. [73], as few as one sensor may indeed be adequate when assessing global movements provided that broader criteria are employed, rather than aiming to highlight a specific movement discrepancy. However, further research is required to further explore this hypothesis. Nonetheless, it is essential to recognise that when using fewer than the maximal available sensors, the best-performing device positions were dependent on the movements assessed by O’Reilly et al. [77, 78]. This is a limitation for real-world applications, as to use the optimal sensor position for each movement would require an adjustment to the anatomical positioning of a sensor, or sensors, to maximise accuracy when conducting a sequential assessment with multiple movements, a potentially time-consuming step. However, it may be plausible to consider a practical compromise by using a single-sensor placement that ensures adequate accuracy when assessing multiple movements, without rigidly adhering to the optimal position for each specific movement. This approach can help alleviate the requirement for time-consuming adjustments to individual sensor positions, making it more feasible and efficient for real-world applications. O’Reilly et al. [77, 78] also conducted intra-study comparisons between binary classifiers, where movements were categorised as either proficient or not proficient, and a multi-class classifier featuring five classes, where the specific deviations from the accepted movement standard were highlighted. Congruent with Kianifar et al. [68], the multi-class classifiers were less accurate than the binary classifiers due to the need to distinguish specific movement errors from the data [77, 78]. However, multi-class classifiers have the potential to be more informative, given that specific movement discrepancies can be detected [77, 78]. Future research to improve the accuracy of multi-class classifiers is therefore warranted. Notably, O’Reilly et al. [77, 78] and Kianifar et al. [68] considered movements that were lower-limb dominant, with the sensor configurations reflecting this. A key consideration of this review is that the accuracies reported in each study may not be comparable if applied to other movements with alternative sensor placements. Moreover, classifiers based on natural movement deviations appear to underperform in comparison to those where movement errors were induced [77]. This highlights a key challenge that may arise when performing movement quality assessments using wearable devices in real-world applications.

While O’Reilly et al. [77, 78] and Kianifar et al. [68] considered the relationship between a reduction in the quantity of sensors and the accuracy of classifiers, each placement location featured a device consisting of more than one type of sensor. This is because each study utilised sensor fusion to provide additional metrics, such as limb orientations and joint angles, which were fed into the respective classification algorithms [68, 77, 78], as was the case with Ghobadi and Esfahani [66], Liu et al. [72], and Tabrizi et al. [82]. Unfortunately, based on the reviewed literature, it is not possible to make comparisons between the use of multi-sensor devices and unimodal sensors in equivalent anatomical positions, nor to consider the use of multiple types of unimodal sensors applied outside of a single device when assessing movement quality. Indeed, the methodological heterogeneity among the included studies prevents conclusions being drawn regarding the potential benefits of using multi-sensor devices over unimodal alternatives. It is speculated that unimodal sensors would not perform as well as multi-sensor devices for identifying movement discrepancies using classifiers, not least because it would only be possible to obtain the orientation data, and subsequently utilise the orientation signal features if sensor combinations exist within one device. In such instances where additional signal features were obtained, classifiers were often very accurate [66, 72, 82], and possibly more so than those with fewer features. However, lower accuracies were also detected using the same signal features under less optimal configurations [68, 77, 78].

Accelerometers were used in isolation in four of the classification studies [62, 69, 70, 84]. Of interest, accelerometers were the only type of sensor used in isolation and most commonly in studies that required classifiers with less complexity. Specifically, Lee et al. [70] and Caporaso and Grazioso [62] used algorithmically simple classifiers based on temporal features of the acceleration signal to detect loss of ground contact during race walking, while Lander et al. [69] and Xu et al. [84] also considered the features of acceleration signals but assessed their correlation with pre-existing signals that corresponded with key assessment criteria. The absence of additional sensors appears to restrict what may be achieved, and while this is not always a barrier when implementing simple classifiers, other studies using additional sensory data have demonstrated greater detail, specificity, and applicability to a wider selection of movements. Indeed, sensor fusion, utilised in six of the classification studies [48, 66, 68, 72, 77, 78, 82] to obtain orientation information as an additional input to the classifier, was reported to be associated with good accuracies.

It is presently unclear as to how influential the specific assessment criteria and movements are on the accuracy of classifiers, meaning it is difficult to definitively state whether additional sensor types within a device can improve classifier accuracy. It was theorised that multi-sensor devices would increase classifier accuracy due to the increase in measurable outputs, though this would only be possible to demonstrate through direct comparisons. However, it is pertinent to note that key a priori decisions, such as the quantity and configuration of sensors, may influence the apparent classifier accuracy and its interaction with protocol-related factors, for example, movements assessed and sensor placement. This is suggested by the comparable binary classifier accuracies presented by Caporaso and Grazioso [62] and Lee et al. [70], who both used a single accelerometer on the lower back, and Kianifar et al. [68], in whose study a single multi-sensor device was worn on the shank. The classifier accuracies in all three studies were determined by comparing against video footage assessed and labelled by an expert [62, 68, 70]. Both Kianifar et al. [68] and Caporaso and Grazioso [62] also employed three-level classifiers, which also indicated comparable accuracies. Notably, Kianifar et al. [68] applied classification methods to a unilateral squat, whereas Caporaso and Grazioso [62] and Lee et al. [70] used classifiers to highlight race-walking infringements. It is feasible that the increased instability that arises with unilateral movements, such as the unilateral squat used by Kianifar et al. [68], could have an influence on the assessment accuracy, offsetting the possible benefits of additional sensors. To reinforce the capability of single-sensor units, Xu et al. [84] presented near perfect classification accuracy when measuring a cycling pedal motion, despite using a single accelerometer. As the motion assessed was constrained to a fixed path, less variation in the movement was possible, thereby potentially simplifying the detection of key signal features.

Traditional feature-based classifiers were a common choice [61, 62, 65, 67, 70, 73], although no indication was given as to why this approach was selected over other classifiers. It is possible that the simplicity of the assessment may be one factor [62, 70] while the benefits of using temporal parameters may be preferred when considering movement sequencing [65]. It does appear, however, that highlighting specific movement discrepancies is lacking in these studies due to a reliance on broad assessment criteria [61, 67, 73]. A further limitation of traditional feature-based classifiers is a reliance on real-world validation through manual assessment, a source of subjectivity and human error [61, 62, 65, 67, 70]. Taken together, it is speculated that supervised machine-learning algorithms are preferable where possible, as they often use the same common signal features for the analysis but can negate much of the human error by removing subjectivity through the addition of algorithm training [66, 68, 72, 77, 78, 81, 82, 84, 85].

While an array of supervised learning methods was considered, SVM was the most widely used. This is unsurprising given that SVM consistently outperforms other classifiers in the reviewed literature, as demonstrated by intra-study comparisons with other methods [68, 72]. Notably, in the only study that utilised multivariate regression [82], SVR, a regression model based on SVM, was shown to have comparable results for quantifying movement quality when evaluated against deep-learning alternatives, namely LSTM and CNN [82]. Indeed, while LSTM marginally outperformed both SVR and CNN models, albeit all models were fit for purpose, SVR may be more optimal than LSTM when using smaller datasets [82]; traditional machine learning algorithms often generate comparable outcomes to deep learning methods under such circumstances [101]. Accordingly, Kianifar et al. [68] specifically highlighted the utility for smaller datasets and high-dimensional data that is characteristic of most of the included classification studies. It is important to recognise, however, that while SVM appears to be preferable in the assessment of movement quality, no machine learning algorithm is uniformly superior under all conditions [102, 103]. Indeed, the wider body of literature illustrates both the benefits and limitations that exist for the numerous machine learning options [102–104]. Hence, it is essential that researchers consider the array of available options when determining the most suitable approach for their intended application.

When employing supervised and unsupervised machine-learning methods, statistical validation methods were generally used in lieu of manual assessments, with KF-CV and LOSOCV being the two primary options. Kianifar et al. [68] utilised and compared both validation methods, with KF-CV providing more accurate results. However, other studies employed LOSOCV with near-perfect accuracy [78], the suitability of which was specifically recommended by O’Reilly et al. [77] for universal classifiers. In contrast, O’Reilly et al. [77] employed LOOCV, as utilised in Masci et. al. [73], to validate a personalised classifier, where the classifier incorporated user-specific details. While this did improve accuracy considerably, the limitation of personal classifiers is the need to adapt to each user, introducing greater time demands and a less user-friendly experience. The most applicable validation method may therefore be dependent on a range of factors, such as the application, sensor positions, and data type. A notable example of this is in the study by Spilz and Munz [81], who implemented a CNN-LSTM layered neural network with two different validation methods, LOSOCV and KF-CV, at different stages of the network architecture.

Whilst unimodal sensor data has been used to assess movement quality [64], sensor-fusion algorithms are particularly prevalent in measurement-based studies. This is likely due to the issues associated with obtaining kinematic data from processing unimodal data, such as integration drift and gimbal lock. Indeed, the application of sensor fusion helps overcome such issues, whereby each sensor compensates the limitations of other sensors [32]. However, sensor fusion is not a complete solution for assessing movement quality when implemented with IMU data as it does not enable the derivation of linear kinematic variables, such as linear displacement and velocity. The measurement of linear motion, therefore, remains reliant on the manipulation of accelerometer data exclusively, which are typically erroneous [1, 32] unless combined with measurements from another measurement system, such as radio-based systems and cameras [32]. Nonetheless, several angular kinematic parameters can be gleaned from the sensor fusion of IMU data alone to highlight specific movement discrepancies [59, 60, 72, 74, 79]. Interestingly, Del Din et al. [64] performed a double integration of accelerometer data to obtain step length, a metric based on linear displacement, reporting that pre-processing using a high-pass Butterworth filter largely addressed the issue of integration drift, though it is pertinent to note that other errors likely persisted [46]. Interestingly, the ICC was excellent between measurement methods, although Del Din et al. [64] acknowledged the limitations of using an instrumented walkway for comparison. Therefore, the findings are likely to be less reliable than studies implementing an optical motion capture system, which is the case for the study by Ahmadi et al. [59] too, where the instrumentation is validated against itself, albeit using optimised configurations. Mitternacht et al. [76] also performed a double integration on the acceleration data captured by the accelerometers to calculate linear position, reducing the influence of integration drift by dividing longer time series data into shorter 0.1 s segments. However, the medial shift estimate was too small to be considered reliable and was reported to a degree of precision that is likely unattainable based on the findings of other research [105, 106]. Unsurprisingly, therefore, the mean medial shift calculated using the IMU was found to be 47% lower than that obtained by the optical motion capture system, a relatively large amount of error. Similarly, Mitternacht et al. [76] and Tulipani et al. [83] integrated gyroscope data to calculate the angular displacement of limb segments. While the results were mostly accurate and reliable in comparison to the gold-standard optical motion capture, it must be reiterated that the degree of tibial tilt observed during the movements in the study by Mitternacht et al. was almost negligible, while Tulipani et al. [83] applied an undisclosed algorithm to, at least in part, overcome the gyroscope deterministic bias offset, rather than simply integrating. Without disclosure of all processing methods, it is not possible to identify what other methods may have influenced the results, thereby limiting interstudy comparisons. It is also important to note that only planar motion was considered, restricting movement to two, rather than three, dimensions, which could be influential on the accuracies reported.

Within the reviewed measurement studies, and indeed all studies included in the review, the Madgwick algorithm [48] was the most frequently used method of sensor fusion. Relative to Kalman and complementary filters, the Madgwick algorithm is still novel. While the reporting of accuracies with each method and the selection of movements is largely heterogeneous, there were no obvious advantages of the Madgwick algorithm identified for achieving accurate measurements in comparison with Kalman and complementary filters. However, both Ahmadi et al. [59] and Shepherd et al. [79] justified their selection of the Madgwick algorithm based on the low-computational demands of the algorithm. Shepherd et al. [79] also identified the suitability of the algorithm when aiming to utilise lower sampling rates and reduce power consumption [59, 79], ideal qualities for incorporation in commercially available wearable devices. While Beange et al. [60] and Shuai et al. [80] did not provide any reasons behind the use of the Kalman filter, Meng et al. [74] highlighted the capability of the complementary filter approach to sensor fusion to overcome drift. However, both the Madgwick algorithm and Kalman filters can also be used to overcome drift, so it is difficult to attribute the selection of a complementary filter on this basis alone. Other possible reasons for the selection of a complementary filter could be the absence of a magnetometer, or simply the ease of implementation [107]. It is also plausible that an undisclosed design feature was utilised as part of the complementary filter to optimise performance for their specific application, given that the authors allude to the use of complementary filters in similar studies also assessing gait [74].

An observed trend is the use of sensor fusion to estimate joint angles, achieved by placing devices on both proximal and distal, or inferior and superior, segments and determining the orientation of each device relative to another [59, 60, 71, 72, 74, 75, 80]. This method allows for the estimation of ROM, a particularly useful metric in both sports and clinical settings. Moreover, orientation data can also be applied relative to fixed coordinate systems. This approach was utilised by Shepherd et al. [79] to assess the forearm angle relative to the ground during a netball shot, and Cortesi et al. [63] to estimate wrist orientation throughout a swimming stroke as part of a more complete motion measurement. Both Shepherd et al. [79] and Cortesi et al. [63] utilise a single device for the measurement of specific components of an activity, which, whilst it may be adequate depending on the application, more complete movement assessments will typically require additional sensors. Indeed, even two sensors, the minimum requirement for estimating joint angles using sensor fusion, has been shown to be insufficient for systemic measurements [60]. Therefore, when using wearable devices, each unit is only appropriate for localised measurements, such as single-limb segments or single joints, thereby necessitating additional sensors to consider broader criteria.

## Conclusion

In conclusion, this systematic review has highlighted some of the key differences between the applications and processing methods associated with the use of unimodal and multi-sensor wearable devices to assess movement quality. Further, the use of multiple devices increases the feasibility of effectively assessing holistic movements, while multi-sensor devices offer the ability to obtain more output metrics. Actions should be taken to further improve measurement accuracy and multi-class classification accuracy, and to translate the systems into affordable, accessible, real-world solutions.

### Supplementary Information

Below is the link to the electronic supplementary material.

Supplementary file1 (XLSX 21 KB)

Supplementary file2 (DOCX 20 KB)

## Declarations

### Availability of data and material

All data generated or analysed during this study are included in this published article and its supplementary information files.
