# Supplementary material for: The Role of Multi-Sensor Measurement in the Assessment of Movement Quality: A Systematic Review
Source: Sports Med. 2023 Sep 12;53(12):2477–504. doi: 10.1007/s40279-023-01905-1 (PMC10687099; doi:10.1007/s40279-023-01905-1)
Supplement: Supplementary file 2 — Supplementary file2 (DOCX 20 KB) [file 40279_2023_1905_MOESM2_ESM.docx]

Online Supplementary Resource 2 – Quality assessment checklist and criteria

| Quality Assessment Criteria | Low (0) | Moderate (1) | High (2) |
| --- | --- | --- | --- |
| 1. Were the research objectives or aims clearly stated? | Research aims not stated | Research aims were not explicitly stated or were insufficiently described | Research aims were clearly described |
|  |  |  |  |
| 1. Was the study design clearly described? | Study design was not described | The study design was mentioned but not clearly described | The study design was clearly described |
|  |  |  |  |
| 1. Was the study population/sample adequately described? | Study sample was not described | Study sample was described but insufficient information was provided | The study sample was clearly described and key details were provided |
|  |  |  |  |
| 1. Were the eligibility criteria specified? | Eligibility criteria were not defined | Eligibility criteria was inappropriate or insufficiently defined | The eligibility criteria were clearly stated |
|  |  |  |  |
| 1. Was the sampling methodology appropriately described? | Sampling methodology was not described | Sampling methodology was limited or insufficiently described | The sampling methodology was clearly described |
|  | | | |
| 1. Was the sample size used justified? | Sample size was not justified | Justification was limited or unsuitable | Adequate justification for the sample size was provided |
|  |  |  |  |
| 1. Did the method description enable accurate replication of the measurement procedures? | The method described was not applicable to the procedure taken | The method was either insufficiently described or the measurement procedures only partially aligned with the method description | The measurement procedures adhered to the method description |
|  |  |  |  |
| 1. Was the assessed movement(s) sufficiently standardised? | Movement was not standardised | Movement was only partially standardised, or the standard lacked detail | A clear standard was provided for the assessed movement |
|  |  |  |  |
| 1. Was the equipment design and set up clearly described? | Equipment and set up were not described | The description of the equipment and set up lacked detail | The equipment and set up was clearly defined |
|  |  |  |  |
| 1. Were sensor locations accurately and clearly described? | Sensor locations were not described | Sensor locations were only visually presented, or the descriptions lacked detail | Sensor locations were clearly described, citing anatomical landmarks where applicable |
|  |  |  |  |
| 1. Was the sensor attachment method clearly described? | Sensor attachment was not described | Sensor attachment was either shown as a figure or the description lacked detail | The sensor attachment method was clearly described |
|  |  |  |  |
| 1. Was the signal/data handling described? | Signal and data handling was not described | Signal and data handling method was not appropriate or was not sufficiently described | Signal and data handling methods were clearly described |
|  |  |  |  |
| 1. Were the main outcomes measured and the related calculations (if applicable) clearly described? | Main outcome measures were not described | Main outcomes, and calculations where applicable, were insufficiently described or were not suitable | Main outcomes and, where applicable, related calculations were clearly described |
|  |  |  |  |
| 1. Was the system compared to an acknowledged gold standard or other acceptable reference standard? | System failed to use a comparative standard | System used a comparative standard but was deemed insufficient or inappropriate | An accepted gold-standard or reference standard was used for comparative purposes |
|  |  |  |  |
| 1. Were measures of reliability/accuracy of the equipment used relative to the reference standard system for the intended outcome reported? | Measures or reliability/accuracy of equipment was not reported or not compared to the reference standard | Measures of reliability/accuracy lacked detail | Measures of reliability/accuracy were provided relative to an acknowledged reference standard |
|  |  |  |  |
| 1. Were the main findings of the study stated? | Main findings were not stated | The findings were not sufficiently stated | The findings of the study were clearly stated and described |
|  |  |  |  |
| 1. Were the statistical tests appropriate? | Statistical tests were not present or were inappropriate | There was an insufficient number of statistical tests or only some of the tests used were relevant | The statistical tests were appropriate |
|  |  |  |  |
| 1. Were limitations of the study clearly described? | Author did not declare any limitations | Limitations were inadequately described or too few were declared relative to the study | Limitations were clearly stated by the author |
